# Supplementary material for: Closed-loop optogenetic control of cell biology enables outcome-driven microscopy
Source: Nat Commun. 2025 Dec 23;17:1087. doi: 10.1038/s41467-025-67848-5 (PMC12852791; doi:10.1038/s41467-025-67848-5)
Supplement: Supplementary file 1 — Supplementary Information [file 41467_2025_67848_MOESM1_ESM.pdf]

Supplementary Information for:

Closed-loop optogenetic control of cell biology enables outcome-driven microscopy

Josiah B. Passmore[†1,2], Alfredo Rates[†1], Jakob Schröder[1], Menno T. P. van Laarhoven[3], Vincent J. W. Hellebrekers[1], Henrik G. van Hoef[1], Antonius J. M. Geurts[1], Wendy van Straaten[1,2], Wilco Nijenhuis[1,2], Florian Berger[1], Carlas S. Smith[3], Ihor Smal[1], Lukas C. Kapitein*[1,2]

[1] Cell Biology, Neurobiology and Biophysics, Department of Biology, Faculty of Science, Utrecht University, Utrecht, The Netherlands.

[2] Centre for Living Technologies, Alliance TU/e, WUR, UU, UMC Utrecht, Utrecht, The Netherlands.

[3] Delft Center for Systems and Control, Delft University of Technology, Delft, The Netherlands.

[†] These authors contributed equally: Josiah B. Passmore, Alfredo Rates.

* Correspondence: l.kapitein@uu.nl.

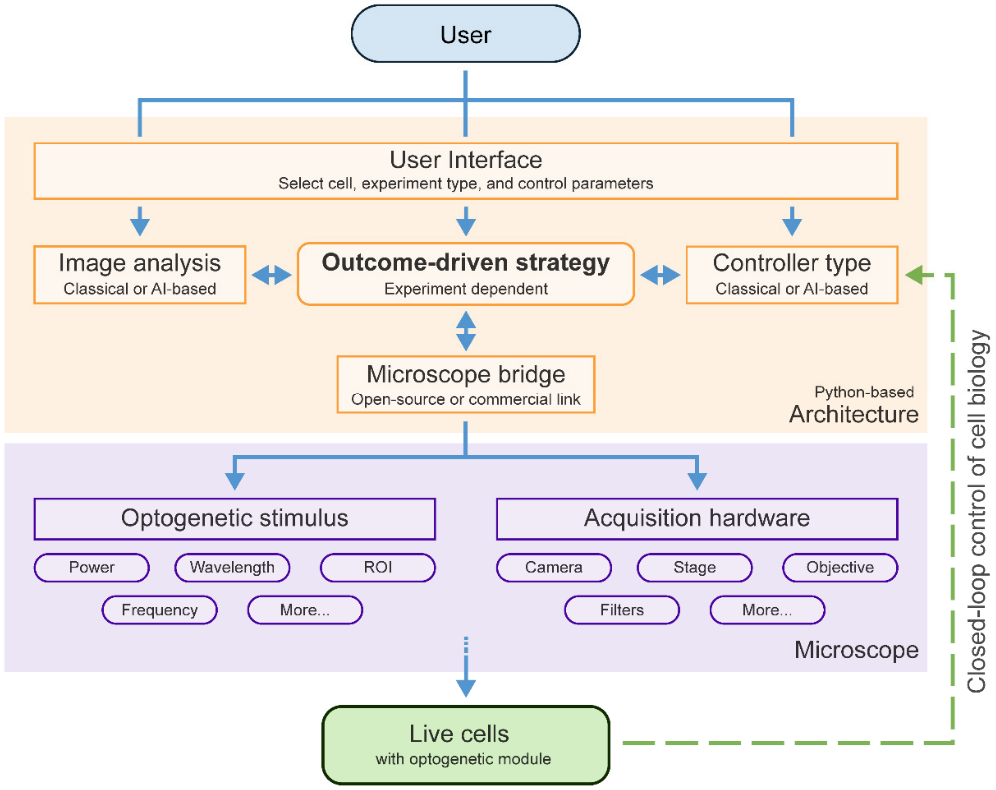

Supplementary Figure 1: A modular platform for smart, outcome-driven microscopy.

A python-based architecture (orange) consisting of user-adjustable modules for microscope bridging, image analysis and feedback control, coordinated by a user-defined outcome-driven strategy. Microscope hardware (purple) is controlled by the architecture bridge, to adjust optogenetic stimulus through a spatial light modulator, change acquisition parameters, and acquire an image. The acquired image of the modified sample is fed back into the architecture at each timepoint to ultimately bring cells to a specific outcome. For a complete description, see Supplementary Text 1.

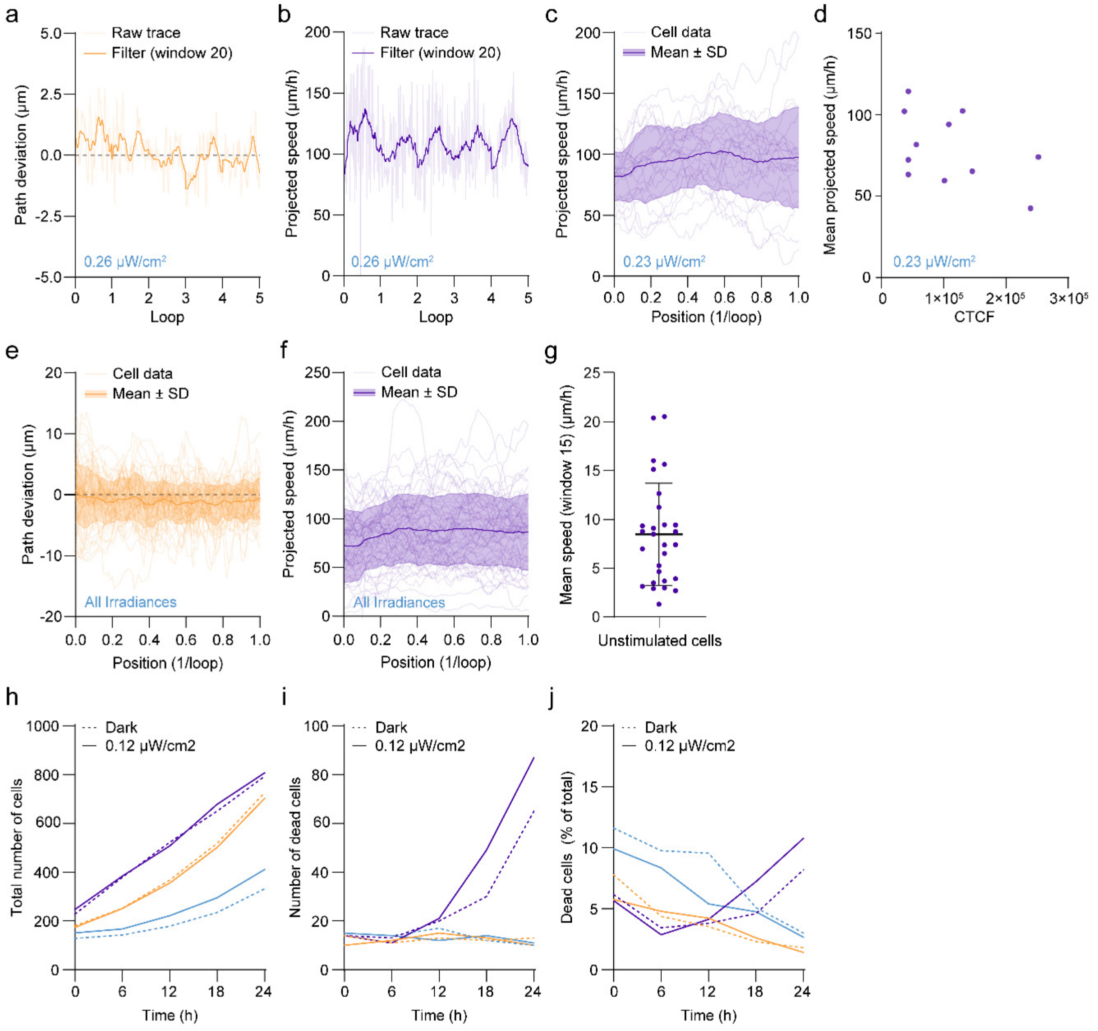

Supplementary Figure 2: Outcome-driven control of directed cell migration.

a) The path deviation of a single HT1080-TIAM cell (from Fig. 1c-d) controlled to a circular path for 5 loops, with a constant irradiance of 0.26 μW/cm^2. **b**) The projected speed of the same cell as in **a**. **c**) The projected speed of HT1080-TIAM cells controlled to circular paths with a constant irradiance of 0.23 μW/cm^2, separated by individual loops. n = 24 cells. Error bands show mean ± standard deviation (SD). **d**) Average projected speed of cells in **c** over the whole controlled circle path, separated by expression level (corrected total cell fluorescence, CTCF). n = 11 cells. **e**) The path deviation of all HT1080-TIAM cells controlled to circular paths, for all irradiances, separated by individual loops. n = 58 cells. Error bands show mean ± standard deviation (SD). **f**) The projected speed of all cells as in **e**. n = 58 cells. Error bands show mean ± standard deviation (SD). **g**) The mean speed of uncontrolled HT1080-TIAM cells (0 μW/cm^2). n = 28 cells from 3 separate coverslips. Error bars show mean ± standard deviation. **h**) The total number of HT1080-TIAM cells per field of view over time for regions kept in the dark (dashed lines), or constantly illuminated with 0.12 μW/cm^2 470 nm light (solid lines). Different colours represent different replicates, n = 3 replicates. **i**) The number of dead (SYTOX Deep Red positive) cells per field of view for the same regions as in **h**. **j**) The percentage of dead cells out of the total number of cells for the same regions as in **h**-**i**. Source data are provided as a Source Data file.

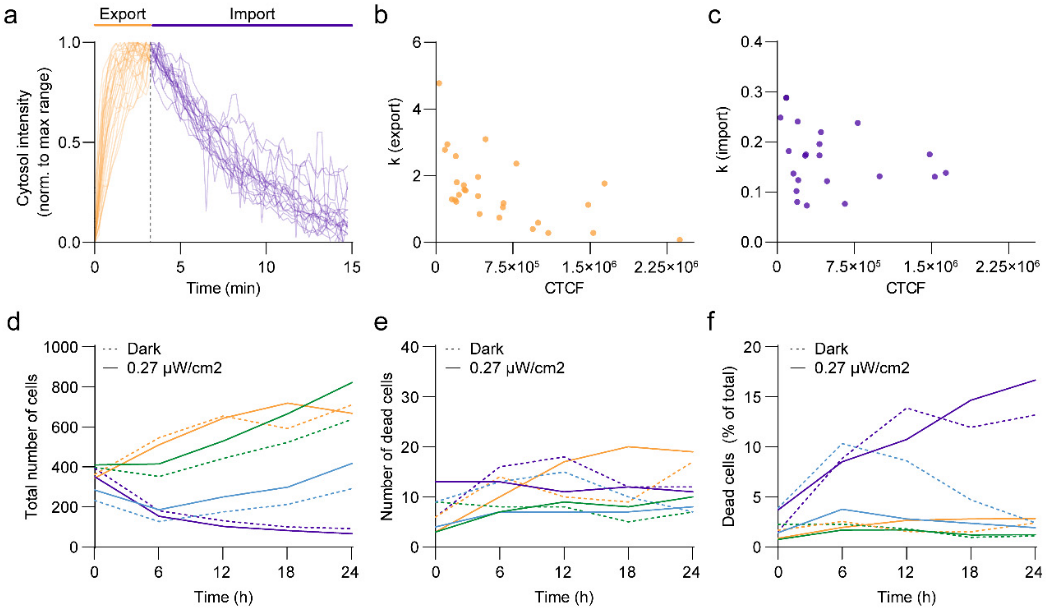

Supplementary Figure 3: Characterisation of U2OS-LEXY cells response to illumination.

a) Cytosolic intensity of U2OS-LEXY cells illuminated with a fixed irradiance of 10.4 µW/cm^2 for 3 min (export), and then kept in the dark for 12 min (import). n = 28 cells. **b**) Decay constant (k) during export for an exponential decay fit to cytosolic intensity during illumination in **a**, separated by expression level (CTCF). **c**) Decay constant (k) during import for an exponential decay fit to cytosolic intensity during the dark state in **a**, separated by CTCF. **d**) The total number of U2OS-LEXY cells per field of view over time for regions kept in the dark (dashed lines), or constantly illuminated with 0.27 µW/cm^2 470 nm light (solid lines). Different colours represent different replicates, n = 4 replicates. **e**) The number of dead (SYTOX Deep Red positive) cells per field of view for the same regions as in **d**. **f**) The percentage of dead cells out of the total number of cells for the same regions as in **d**-**e**. Source data are provided as a Source Data file.

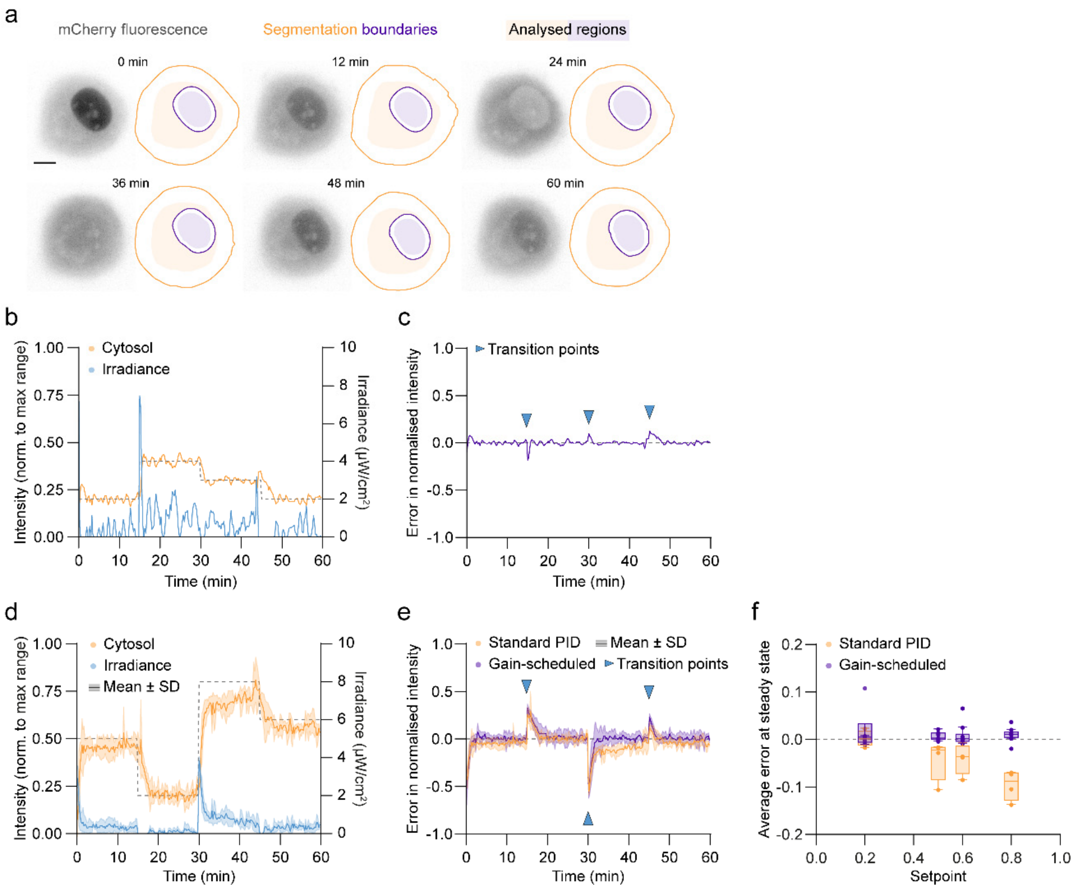

Supplementary Figure 4: A gain-scheduled PID controller outperforms a standard controller over the full operating range.

a) A U2OS-LEXY cell (grey) controlled using outcome-driven microscopy to achieve predefined cytosol intensities, showing segmentation of the cytosol (orange) and the nucleus (purple), and regions analysed for control. Scale bar: 10 µm. **b**) Normalised cytosol intensity and irradiance of the cell in **a** controlled to various setpoints (dashed line) using a standard PID controller. **c**) Error in normalised intensity for the cell in **a**-**b**, with blue arrowheads to denote changes of setpoint. **d**) Normalised cytosol intensities and irradiances of multiple cells over time, controlled by a standard PID controller over the whole operating range. n = 4 cells. Error bands show mean ± standard deviation (SD). **e**) The error in normalised intensity (intensity–setpoint) for cells guided to the same setpoints as in c, with the standard and gain-scheduled PID controllers. Transition points (changes of setpoint) are shown with blue arrowheads. n = 4 cells (standard), n = 10 cells (gain-scheduled). Error bands show mean ± standard deviation (SD). **f**) The distributions of average error at steady state for each setpoint, for both standard and gain-scheduled PID controllers. n = 4 cells (standard), 10 cells (gain-scheduled). Source data are provided as a Source Data file.

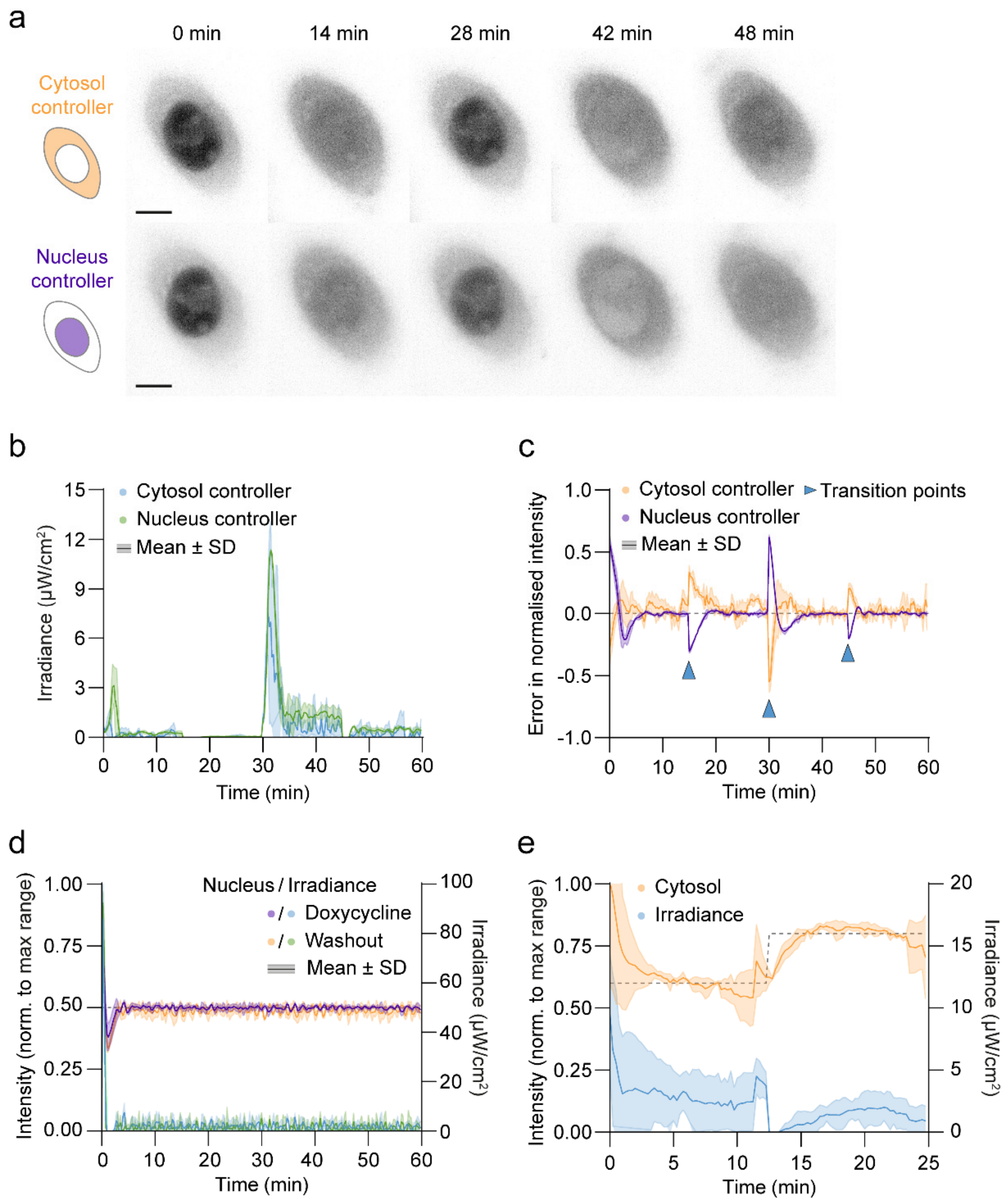

Supplementary Figure 5: Outcome-driven control of nucleus intensity.

a) Snapshots of the U2OS-LEXY cell controlled with both cytosol and nucleus controllers to achieve predefined cytosol and nucleus intensities shown in Fig. **3d**. Scale bars: 10 µm. **b**) Irradiance levels for the cell controlled in Fig. **3d**, for both the cytosol and nucleus controller. n = 3 runs for each controller. Error bands show mean ± standard deviation (SD). **c**) Error in normalised intensity for the control of cytosol and nucleus intensity in Fig. 3d. n = 3 runs for each controller. Error bands show mean ± standard deviation (SD). **d**) Cells controlled to the same normalised nucleus intensity in the presence of, and after washout of doxycycline. n = 3 cells for each condition. Error bands show mean ± standard deviation (SD). **e**) COS-7 cells transiently transfected with NLS-mCherry-LEXY and controlled to the same normalised nucleus intensity. n = 3 cells. Error bands show mean ± standard deviation (SD). Source data are provided as a Source Data file.

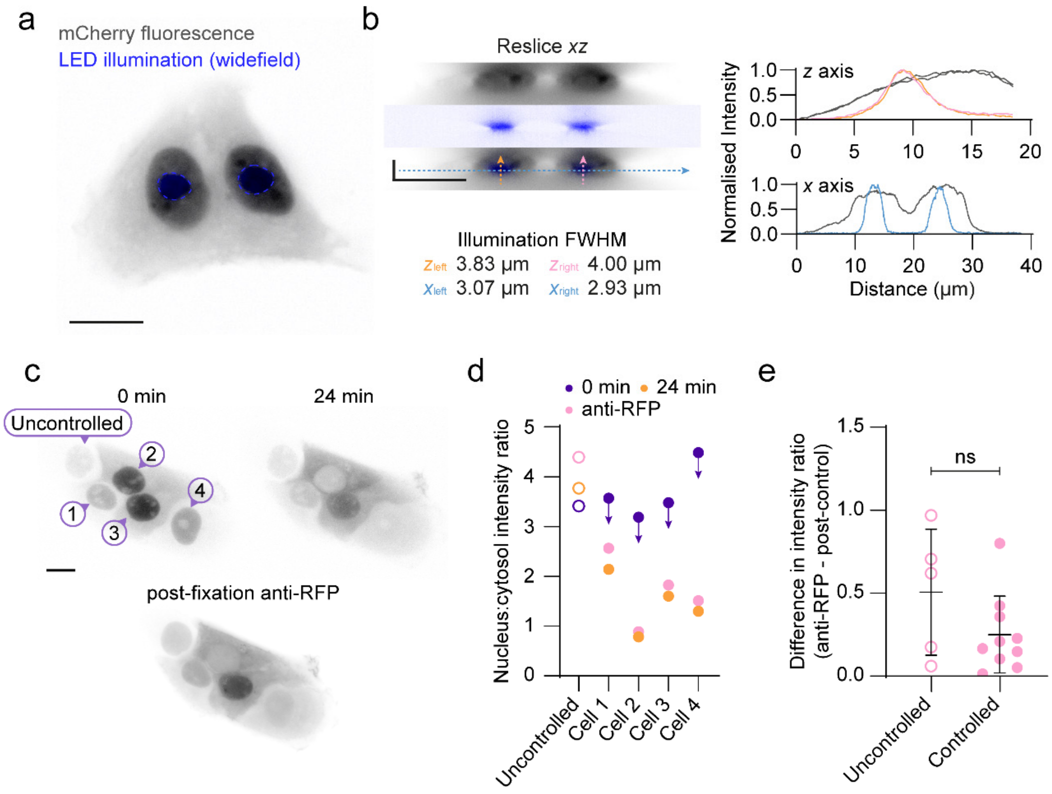

Supplementary Figure 6: Validation experiments for multi-cell control of nucleocytoplasmic transport.

a) Single slice of a widefield z-stack of U2OS-LEXY cells illuminated in a region in the centre of the cell with 470-nm LED. Scale bar: 10 µm. **b**) Reslice of the widefield z-stack shown in **a**, with dashed lines indicating the position of line scans. Corresponding intensity profiles of line scans shown for both LED illumination (indicated colour) and mCherry fluorescence (grey), with quantification of the Full Width at Half Maximum (FWHM) of the LED illumination. Scale bars: 10 µm. **c**) Four U2OS-LEXY cells controlled to different nuclear intensities, fixed on-stage and immunolabelled for RFP (to visualise the NLS-mCherry-LEXY shuttled protein). **d**) Quantification of nucleus:cytosol intensity ratio for the cells shown in **c**. **e**) The difference in nucleus:cytosol intensity ratio between immunofluorescence against RFP, and post-control (immediately before fixation), for both uncontrolled and controlled cells. n = 5 cells (uncontrolled), n = 10 cells (controlled). ns = not significant, P = 0.2544 (Mann–Whitney U test, two-sided). Error bars show mean ± standard deviation. Source data are provided as a Source Data file.

Laser	BP Filter	Detector	Fluorochrome	Voltage	Threshold
488		FSC		1	5000
488		SSC		300	
488	530/30	B	mVenus	395	
561	582/15	D	TagRFP-t	448	

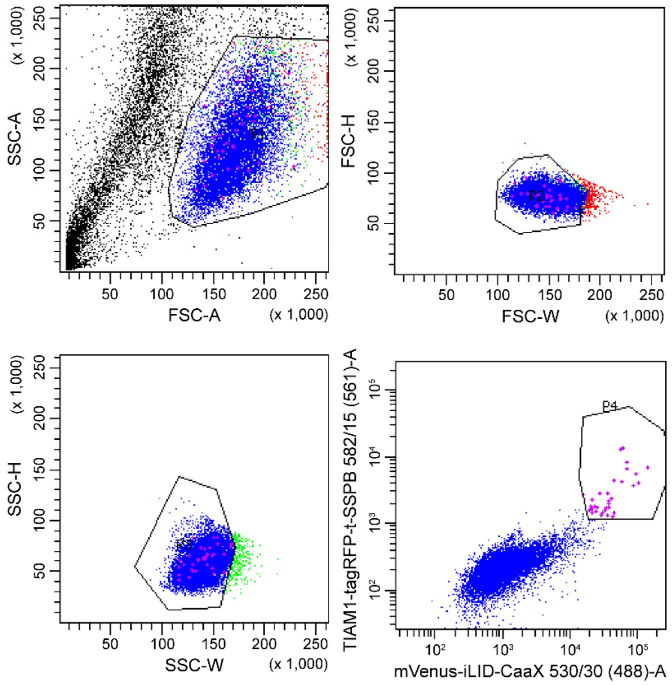

Sort Settings

**Nozzle size (µm)**	85
**Pressure (psi)**	45
**Drop delay**	39.23
**Sort mode**	4-way purity
**Sort device**	2tube
**Amplitude**	16.1
**Frequency**	69.1
**Drop 1**	182
**Gap**	9
**Voltage**	4500

Sorted

**Total cells**	48632
**P4**	1147
**Total time**	00:46:59

Supplementary Figure 7: Gating strategy for isolation of double-positive cells by FACS.

Cells were analysed on a FACSAria™ Fusion Flow Cytometer (BD Biosciences). Gates were applied to select the primary cell population using Forward Scatter-Area (FSC-A) vs Side Scatter-Area (SSC-A), select singlets using Forward Scatter-Width (FSC-W) vs Forward Scatter-Height (FSC-H) and Side Scatter-Width (SSC-W) vs Side Scatter-Height (SSC-H), and finally select cells positive for both mVenus (530/30, 488 nm laser) and TagRFP-t (582/15, 561 nm laser). The mVenus/TagRFP-t population (P4, magenta) was collected for further experiments. Instrument settings and sort parameters are indicated.

Supplementary Notes for:

Closed-loop optogenetic control of cell biology enables outcome-driven microscopy

Josiah B. Passmore[1,2†], Alfredo Rates[1†], Jakob Schröder[1], Menno T. P. van Laarhoven[3], Vincent J. W. Hellebrekers[1], Henrik G. van Hoef[1], Antonius J. M. Geurts[1], Wendy van Straaten[1,2], Wilco Nijenhuis[1,2], Florian Berger[1], Carlas S. Smith[3], Ihor Smal[1], Lukas C. Kapitein[1,2]

[1]Cell Biology, Neurobiology and Biophysics, Department of Biology, Faculty of Science, Utrecht University, Utrecht, The Netherlands.
[2]Centre for Living Technologies, Alliance TU/e, WUR, UU, UMC Utrecht, Utrecht, The Netherlands.
[3]Delft Center for Systems and Control, Delft University of Technology, Delft,The Netherlands.

Corresponding author: l.kapitein@uu.nl;
[†]These authors contributed equally: Josiah B. Passmore, Alfredo Rates.

Supplementary Note 1: Modular Python platform for smart microscopy

We developed a Python-based platform (version 3.9.13) to perform automated, outcome-driven experiments. Our platform is organized into multiple modules, as described in Supplementary Figure 1. In this section, we describe the general structure of the platform, followed by a more detailed description of each module. We also describe a small example of how to run an experiment from the user perspective, followed by future improvements and modifications to the platform.

1.1 Modularity and structure

The abstract architecture is as described in Supplementary Figure 8. The user interacts with the platform through a `main.py` script and a graphical user interface (GUI). The `main` connects to the rest of the modules, including the GUI, and runs three parallel, asynchronous loops. The three loops consist of the *interface loop*, the *analysis loop*, and the *control loop*. These loops connect to the independent modules as shown in Supplementary Figure 8.

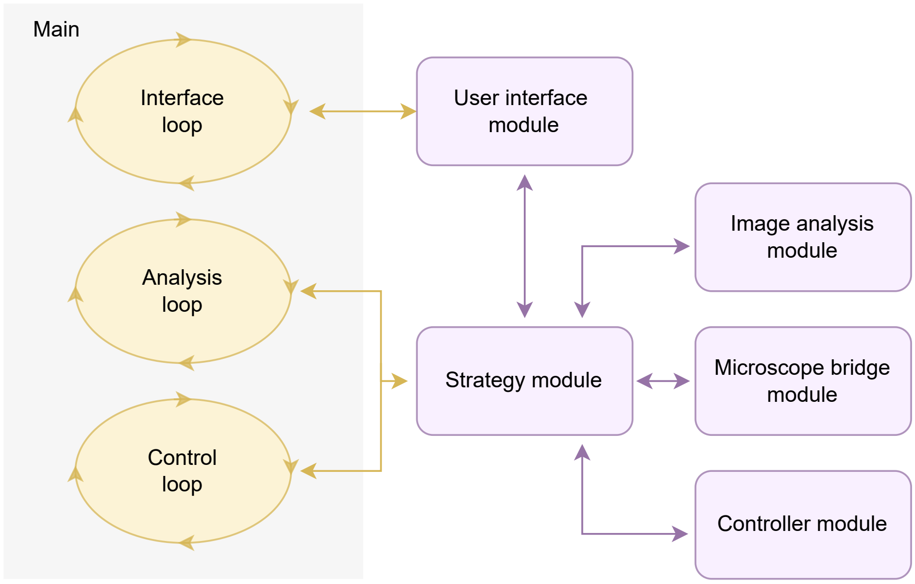

Supplementary Figure 8: Module workflow showing details of the python-based architecture. The `main.py` script with three internal parallel loops (orange) is used to coordinate the user-adjustable modules (purple).

The modules are imported to the `main` as external libraries based on the user input. Each module is structured as a class with an *abstract class* as a parent, meaning that they must have a list of functions and variables in order to communicate correctly with the rest of the platform. In case the user needs to develop a custom module, it must include these functions and variables as well.

When running an experiment, the platform exports four main results. The first result is the time series acquisition, including channels and z-stacks, if previously defined. In addition, the platform exports the cell segmentation for each time step, the controller data (*e.g.*, error, setpoint, laser power) as a `.csv` file, and the metadata of the experiment as a `.json` file.

1.2 Strategy module

The center module of our platform is the (outcome-driven) strategy, *i.e.*, the experiment to be performed. The strategy module can be adapted to any kind of smart microscopy experiment, either outcome-driven, event-driven, or pre-programmed. Here, we show two outcome-driven strategies using optogenetics: one to guide a migratory cell and the other to control the cytosolic and nuclear levels of a cell.

The functions and parameters a strategy module must have, based on their *abstract (template) class* parent, are:

1. `process_step()`: function that connects to the image analysis module and extracts the correct information from the image and segmentation for the closed-loop control.
2. `controller_step()`: function that connects to the controller module and converts the abstract output of the controller to the type of output that will be sent to the microscope.
3. `write_data()`: function to export data to a `.csv` file. The exported data is updated at every step and the variables exported depend on the experiment.
4. `set_parameters()`: function to change internal parameters of the strategy module by the main.
5. `processed_image` and `mod_image`: variables of the segmentation (*i.e.*, output of the image analysis module) and the image to be sent to the modulator, respectively. These variables are retrieved by the `main`, especially to visualize in the GUI.

For the two outcome-driven strategies presented in the main text, the functions `process_step()` and `controller_step()` take different roles. These functions are called during the analysis and controller loops in `main`, respectively. We describe their specific roles in Supplementary Table 1.

	**Cytosol concentration**	**Cell migration**
`process_step()`	Used to obtain the cell segmentation from a given region.	Used to get directed illumination and for the Active Avoidance of multiple cells.
`controller_step()`	Used to obtain the segmentation of the cytosol and nucleus, erode the edges of each mask to avoid overlap, and calculate the concentration of nucleus and cytosol.	Used to update the setpoint if needed and obtain new irradiance from the controller.

Supplementary Table 1: The roles of the main functions in the strategy module (`process_step()` and `controller_step()`) for controlling cytosol concentration and cell migration.

In terms of platform, the user can choose any strategy without restrictions. The user interface only considers the inputs relevant to the active module, and the other modules are not affected by the strategy used. Furthermore, the strategy module is responsible for and sets the rest of the modules, with the exception of the graphical

interface module. If the user wants to develop a new strategy for a tailored experiment, they must build a new strategy module.

However, it is worth noting that our platform does not yet perfectly separate the strategy module from the microscope bridge module explained below. If a smart microscopy strategy requires a completely different experiment design, the microscope bridge must also be adapted. We develop this idea further in Section 1.10.

1.3 Image analysis module

The image analysis module produces a binary mask based on the microscope image. The binary mask represents an area of interest and can be a single cell, a nucleus, a cluster of cells, or sub-cellular structures. For the two outcome-driven examples given, we segment a whole cell for induced cell migration and the cytosol and nucleus of a cell for cytosol concentration.

Although the image analysis module is independent and can be replaced with different segmentation approaches, we used the same algorithm, the Segment Anything Model (SAM), for both examples as the results outperformed any other algorithm that we tested. The segmentation is described in detail in Supplementary Note 2:.

The *abstract class* for segmentation defines the following mandatory functions:

1. `setImage()`: function to define the image to segment. This function is typically called after taking an image with the microscope.
2. `setup()`: function to define a region of interest within the image for the segmentation, *e.g.*, where the cell is located. When this function is ignored, the whole image will be used as the region of interest.
3. `run()` and `getResult()`: functions to run the segmentation and to retrieve the result of the segmentation, respectively.

1.4 Controller module

While the strategy module processes the information and decides what variable is optimized, the controller module is where the optimization occurs. The controller module has a single mandatory function, `step()`. On every step in time, this function calculates the signal (*e.g.*, irradiance, area of illumination, frequency) to be sent to the biological sample on the next step. The controller module can be design around any type of controller, from classical linear controllers, to model-based controller, to AI-based controllers. Although the type of controller depends on the experiment, the control module is independent of how the input to the controller (*i.e.*, the signal to be controlled) is obtained. Thus, controller modules are in principle exchangeable.

For the two outcome-driven experiments presented here, we developed two controllers; one for the migration experiment and the other for the cytosol concentration experiment.

1.4.1 Cell migration control

For the cell migration experiment, the `step()` function calculates the *area* of the cell to illuminate with blue light based on the position of the cell and the pre-defined path

to follow, always illuminating the edge of the membrane to induce migration in the selected direction (see Figure 1b in the main text). The input then is the raw image of the cell and the (initial) position of the cell.

1.4.2 Cytosol concentration

In turn, for the cytosol concentration experiment, the `step()` function calculates the *irradiance* of the blue light to reach a certain concentration. We use a Proportional-Integral-Derivative (PID) controller to obtain the optimal irradiance value to drive the system to a given setpoint. The input for the controller are the estimated and desired concentration. The controller design is described in detail in Supplementary Note 3:.

1.5 Microscope bridge module

The bridge module is the independent module in charge of communicating with the microscope. Thus, the platform controls the hardware and the time series acquisition through the bridge module. For the two outcome-driven examples given, the bridge module requires access to the camera, the light modulator device, and the blue light LED. Still, for a different experiment, the bridge module may have access to other hardware components such as stage, objectives, or temperature.

For the presented experiments, we controlled a Nikon Ti dual-turret inverted microscope using the Micromanager software [1], a versatile open-software program that allows to control many microscopes from different brands. To communicate with Micromanager, we built a bridge module based on `Pycromanager` [2], a Python-based library that allows easy communication with Micromanager.

Thanks to the modularity design of our platform, the bridge module can be exchanged to run exactly the same experiment in different systems. A bridge module can be developed to use other communication libraries, such as `pymmcore-plus` [3], `navigate` [4], or `ImSwitch` [5]. Furthermore, we are working on developing bridge modules for other (commercial) microscopes. This is described more in depth in Section 1.10.

For any microscope, a bridge module must have the following functions and parameters:

1. `live_image()`: function to take a live image or *snap* from the microscope.
2. `set_modulation()`: function to set a binary mask into the modulator device for the blue-light illumination. This function can be adapted to systems without modulators, *e.g.*, setting a scanning area for a scanning microscope.
3. `run_acquisition()`: function to run the multi-dimensional acquisition. This starts the time-lapse acquisition with the given channels, z-stack, and positions.
4. `shutdown()`: function to start the protocol to turn off the hardware after the experiment, if needed.
5. `Acq_image`: the current image of the multi-dimensional acquisition, once it is running.
6. `modulator_dict`: a dictionary with the information for the modulator device, namely the binary mask and the LED power.

7. `im_height` and `im_width`: are parameters retrieved by main, representing the height and width of the camera images, in pixels.
8. `mod_height` and `mod_width`: are parameters retrieved by main, representing the height and width of the modulator, in pixels.
9. `acq_path` and `acq_name`: are the folder and file names for the resulting time-lapse images. These variables are defined by the user in the main.
10. `image_ready` and `modulator_ready`: are boolean variables observed in main to know if a new image is retrieved or if the modulator image was correctly assigned, respectively.
11. `running`: variable to notify the main that the multi-dimensional acquisition is currently running.

1.5.1 Irradiance calibration

To achieve reproducible control of irradiance-dependent processes, the irradiance at the sample plane was characterized for the Nikon Ti microscope. In this way, the commands generated from the controller are decoupled from the microscope on which the experiment is running. We used a ThorLabs PM400 power meter with a S121C head placed at the sample plane to measure the irradiance, whilst the commands to the 470 nm illumination LED was varied. We also included optical density filters to reduce the total irradiance, and calibrate for each filter. The results of this calibration experiment is presented in Supplementary Figure 9. For each calibration curve, a fitted polynomial was used to convert irradiance targets from the controller to commands for the LED.

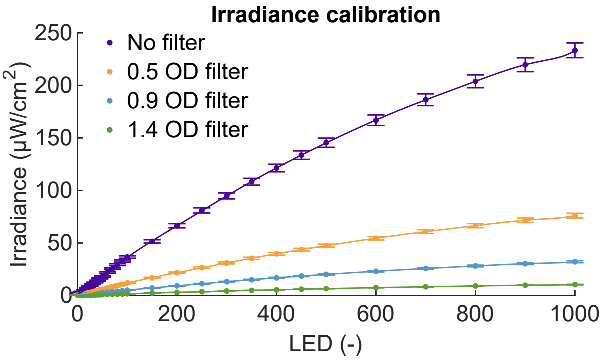

Supplementary Figure 9: **Calibration of LED commands to irradiance.** The measured irradiance at different LED commands with various optical filters placed in the light path. Error bars show measured value ± 3% (reported accuracy of the power meter used). An 11-order polynomial is fitted to the data. Source data are provided as a Source Data file.

1.6 Graphical user interface module

We developed a graphical user interface (GUI) based on the `Tkinter` python library [6]. The user interface is shown in Supplementary Figure 10. The GUI has three main functionalities, to **show** the microscope pictures and segmentation, to **interact** with the user via clicks, and to **start** the time series acquisition. The GUI communicates with the segmentation and bridge module to show the pictures and segmentation, obtaining the images from them. When a user interacts with the GUI, the GUI sends the commands to the strategy module. Once the user wants to begin the time series, the *Acquire* button sends the command to the bridge module.

To communicate with the other modules and the `main`, we import an additional python file called `globVars.py` on every module. This file has global variables that are read from multiple classes.

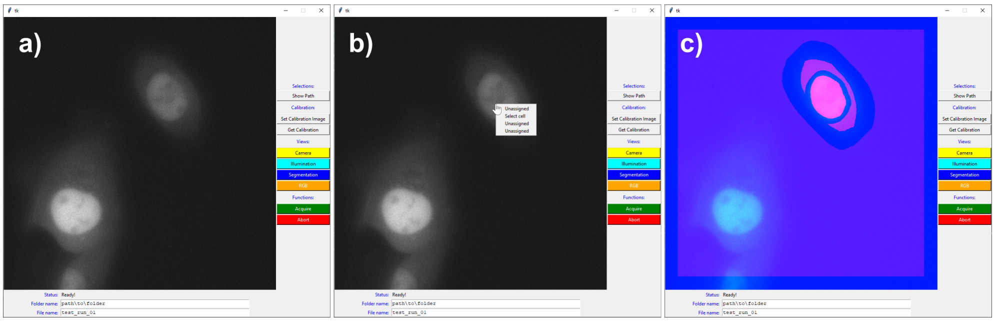

Supplementary Figure 10: **Graphical user interface in three different states.** **a**) Camera image after initialization of the software. with options displayed on the right. **b**) The drop-down menu displayed when a chosen cell is right-clicked. **c**) The RGB visualization showing an overlay of the camera image, cell segmentation, and illumination pattern.

1.7 User steps

Here, we give a brief step-by-step for a user to run an outcome-driven experiment using our platform. We consider a case where the user wants to run an experiment for control of cytosolic concentration. After finding and focusing on a single cell, the user needs to go through the following steps:

1. First, the user needs to **define the input variables** in the `inputs.yaml` file. The input variables include the strategy and bridge module to be used, the variable to be controlled by the PID (cytosol or nucleus concentration), the name of the file and folder, and acquisition parameters such as exposure time, acquisition channel, and time series details.
2. With the input variables defined, the user must run the `main`, which opens the GUI. The user then needs to **select the cell** to be controlled, right-clicking the GUI and selecting the segmentation option from the drop menu.

3. Before running the experiment, the user will need to **calibrate the experiment**. The calibration depends on the experiment. For cytosol concentration experiments, we recommend running an initial measurement with a high setpoint to measure the total dynamic range of the cell.
4. Once the user is satisfied with the cell segmentation and calibration, they can start the acquisition by **pressing the `Acquire` button**. In our modules, this will trigger the multi-dimensional acquisition of Micromanager.

1.8 Workflow of threads and modules

In this section, we describe the main threads of the platform, along with the existing exchangeable modules. To have a better visual representation of how these modules communicate in a threading environment, we design a diagram with the workflow, shown in Supplementary Figure 11.

In Supplementary Figure 11, we show how the three threads communicate with each other based on which module is active. The pipeline workflow starts with the user defining the main parameters and selecting the cell to work with. In order to do so, there is a recurrent loop where the control thread takes a snap with the camera and the analysis thread segments the cell around the point the user selects. Once satisfied with the segmentation, the user can start the time series. After every new acquisition, the image is sent to the analysis thread in order to adjust the segmentation and take a new step in the controller. This will change the optogenetic illumination in the control thread and the interface visualization in the main thread.

This representation of the workflow illustrates how the modules work together and also how modules can be exchanged or added to change the experiment design. For example, for migration experiments, adding a stage control module after changing the illumination pattern would allow us to track a migratory cell, in case the cell goes out of the FOV. Alternatively, a new analysis block can be added to decide to change the control from one cell to another if the cell being controlled stop responding or dies.

1.9 Processing time

To study the limits of our platform, we perform a timing testing of an experiment. Because the processing time was expected to escalate with the number of cells being controlled, we measured the experiment time while controlling the cytosol concentration of an increasing number of cells. We measured the time taken in our experiment setup for the analysis loop, the controller loop, and the total processing time. These experiments were conducted on a computer equipped with an Intel© Xeon w3-2435 processor (3.10 GHz), 128 GB of RAM, and an NVIDIA RTX 4000 GPU. The system operated on a 64-bit Windows OS.

The data collected is presented in Supplementary Table 2. From the data, we see that the total processing time scale linearly with the number of cells, thus having a $\mathcal{O}(n)$ complexity. Note that the total processing time is larger than the sum of the analysis and the controller time as these do not consider hardware control.

The platform finds its limit when the processing time is too long with respect to the time step of the acquisition. Because we apply a constant illumination to the cells

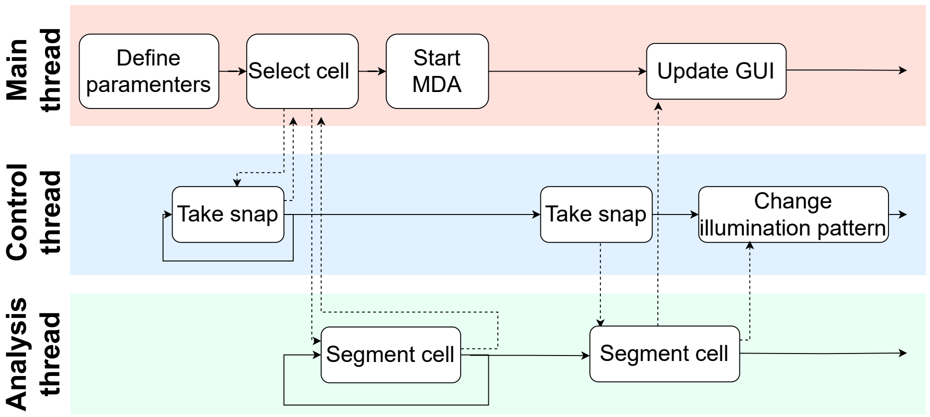

Supplementary Figure 11: **The sequence of events driven by the three parallel threads.** The initial loop continues until the user selects one or more cells to segment and control, and the main loop uses continually updates the illumination pattern based on the segmentation output and control. When the user decides to start the multi-dimension acquisition the two loops connect.

**Number of cells**	**Analysis loop (s)**		**Controller loop (s)**		**Full loop (s)**	
	avg.	std. dev.	avg.	std. dev.	avg.	std. dev.
1	0.978	0.049	0.020	<0.001	2.405	0.321
3	1.331	0.085	0.055	<0.001	3.142	0.279
5	1.492	0.074	0.091	<0.001	2.859	0.256
10	1.884	0.057	0.182	0.003	3.707	0.296
20	2.794	0.115	0.394	0.008	4.950	0.335
50	6.810	0.271	1.061	0.012	9.877	0.344

Supplementary Table 2: The processing time in seconds (s) for the main loops of the platform to control an increasing number of cells.

between snaps in our experiment, if the processing takes too long, the input is applied too late and the controller is not optimal. We found for our case that this ceiling is reached when the processing time takes longer than half of the time step. For the example shown controlling cytosol concentration with a 15 second interval, the ceiling is around 30 cells. Although other outcome-driven implementations may not have this limitation, another hard limitation is when the processing time is equal or longer than the time step. If that were the case in our experiment, then the limit would be around 70 cells.

We estimate that the base computing time in our examples is mainly due to segmentation, as it is the most computationally demanding step on the platform. If high speed is needed, a less intensive segmentation algorithm such as thresholding can reduce the base time to milliseconds. Nevertheless, we believe segmentation is not the reason of the scaling of the computing time, as the segmentation step is done only once but with more region of interests. We attribute this instead to image processing,

like applying the masks to the microscope image, as these are done iteratively. The algorithm can possibly be optimized to be more time efficient, but the processing time can also be shortened if the image has lower bit rate or if binning is applied. Finally, although our computer is a high-end machine, it is still a desktop computer. We believe that the processing time can be drastically reduced if instead the data is transferred to a server or a local processing machine in real time.

1.10 Future modifications

The advantage of modularity is not only that modules are exchangeable, but also that the platform is easily upgraded and improved. Among the improvements planned to the platform, we first intend to **separate further the bridge module with the strategy module**. In particular, the platform is structured around the multi-dimensional acquisition process of Micromanager. This structure is ideal for our outcome-driven experiments and can be translated to other microscopes easily, but it limits experiments to having a constant acquisition configuration, such as time step and number of Z-stack layers. To make our platform flexible, we plan to follow the structure offered by the `pymmcore-plus` library. `pymmcore-plus` is an alternative to `Pycromanager` to control Micromanager-compatible hardware, and is based around a *queue of events* instead of the multi-dimensional acquisition, *i.e.*, a list of orders to give to the microscope.

Furthermore, we plan to **make a new interface** based on the `Napari` library [7]. `Napari` is an extensive visualization library, widely used for microscope data. This new interface offers more functionalities and interaction with the user, and it is also compatible with `pymmcore-plus`.

Additionally, we intend to **develop more bridge modules** to control microscopes that are not compatible with Micromanager. We are especially interested in extending our platform to commercial microscopes such as Zeiss and Leica. For Zeiss microscopes, we plan to use the newly available API functionality, and the LAS X software of Leica also offers opportunities for external control.

Finally, we are aware that many solutions, tools, and architectures are being developed for smart microscopy. For our platform to be compatible with multiple (new and incoming) software and hardware, we intend to **make our bridges portable** with solutions such as `Docker` [8]. `Docker` allows to export *containers*, which are pieces of software that can be used independently of the set of libraries, language, or the computer‘s operative system. Thus, our platform will be compatible with modules created by external community members even if they are not based on Python.

Supplementary Note 2: Image segmentation

As mentioned previously, we used the Segment Anything Model (SAM) for cell segmentation. The Segment Anything project rephrased instance segmentation into a general task by leveraging the masked auto-encoder (MAE) [9] together with an extensive dataset of "over 1 billion masks on 11M licensed and privacy respecting images" [10].

Unsupervised learning strategies, such as MAE's masked-data reconstruction, are shown to generalize well to unseen data [9],[11]. SAM adopted a pre-trained MAE as its image encoder, which creates an implicit description (embedding) of the image, and then queries this, based on prompts (points or bounding boxes), that are then transformed by a mask decoder to produce segmentations. SAM introduced and trained this mask-decoder, together with components to encode the different types of prompts. *A priori*, mask-decoding is a relatively simple task, and, especially considering the magnitude of the training dataset, it is unsurprising that this also generalizes well.

This motivates the use of SAM to a wide variety of image types, including phase-contrast or confocal microscopy data. In practice, SAM was sufficiently accurate and robust for our experiments, such that fine-tuning by transfer-learning was unnecessary for our experiments.

2.1 Custom Interface

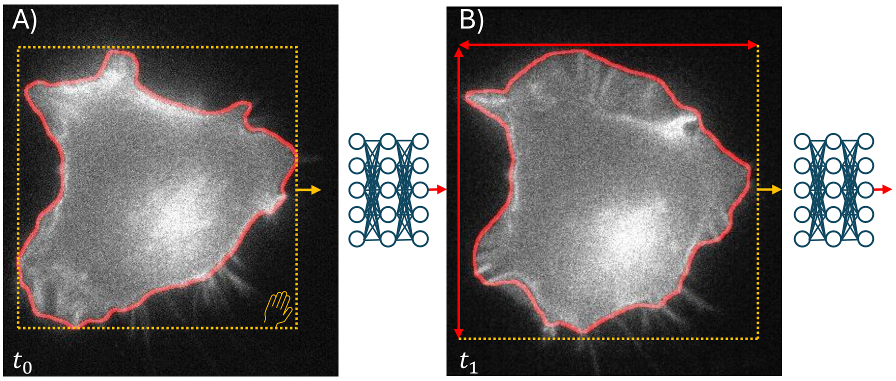

Supplementary Figure 12: **Image segmentation and tracking of a migrating cell.** **A**) A bounding box created of a fixed size after selection of a cell by the user. This bounding box is used as a prompt in SAM. A new bounding box is extracted based on the segmentation output. **B**) After a new image is acquired, the new image is embedded in SAM and prompted using the new bounding box. This process is iterated until the end of the experiment.

SAM and its interface were designed for 1024×1024 8-bit RGB images. Our (8- or 16- bit) single-channel time series are therefore not natively supported. A single frame is therefore re-scaled, repeated 3 times for “RGB” and normalized. The pre-processed image is fed to the model through the (private) tensor functions, bypassing the interface, to preserve the 16-bit accuracy. To initialize the segmentation pipeline, a user specifies the object(s) of interest using a bounding box(es) (Supplementary Figure 12A) in the UI (See section Supplementary Note 1:). Bounding box prompts were chosen as they are inherently more descriptive then a single point (and a single point did not produce robust results during testing). Optionally, the bounding box can be updated to fit the result of the segmentation (Supplementary Figure 12B). The bounding box is re-used for the next image in the sequence and these steps are iterated during the remainder of the experiment.

During the migrating cell experiments we updated the bounding box using the segmentation outputs. For the nuclear import/export experiments, the bounding box was unchanged during the recordings. This is motivated by the negligible movement of the cells during a recording session, while also preventing undetected problematic timepoints (at which nuclear and cytosol have nearly equivalent intensities) from affecting the bounding box. Problematic timepoints are detected by nucleus size (more than ±20% change with regards to the previous nucleus), nucleus position (less than 85% overlap with previous nucleus) or morphology (circularity smaller than 0.85). For problematic timepoints, the segmentation results are replaced by results from the previous timepoint to perform image analysis and control (see Supplementary Figure 13).

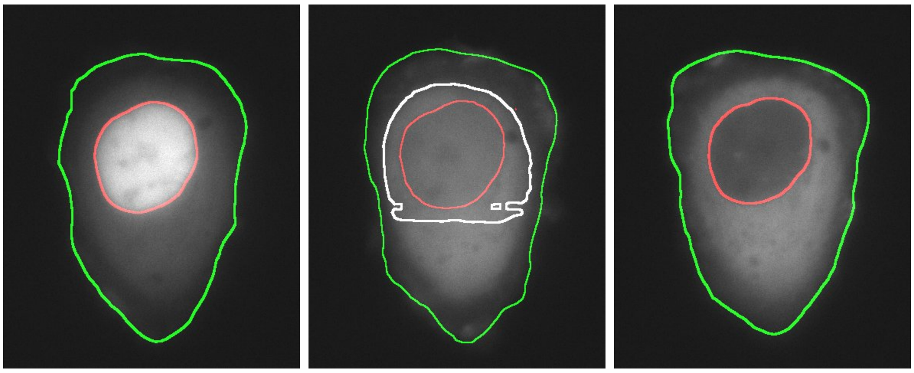

Supplementary Figure 13: **Image segmentation with filtering for LEXY with default, equivalent and inverted Cytosol-Nucleus concentrations.** The outline of the cytosol segmentation is shown in green and the nucleus segmentation in red. For the middle image, the unfiltered nucleus segmentation is shown in white.

Supplementary Note 3: Controller design for nucleocytoplasmic transport

To actively control the nucleocytoplasmic concentrations, we aim to derive a target illumination intensity from a measured fluorescence intensity, with robustness against cell-cell variations. As mentioned, we employed a PID controller to illustrate the efficacy of simple techniques [12]. Although PID control is rooted in linear system theory, its versatility leads to straightforward adaptations to deal with the nonlinear dynamics in nucleocytoplasmic transport.

We first derive a first-principle model for the measured intensity dynamics, which is then used to find suitable PID parameters. The nucleic and cytosolic intensity measurements $i_\bullet$ are quantified in terms of the saturation point s for ease of user interaction. The saturation is computed from the minimum and maximum achievable concentration $i_{\bullet,\text{min}}$ and $i_{\bullet,\text{max}}$,

$$s = n(i_\bullet) = \frac{i_\bullet - i_{\bullet,\text{min}}}{i_{\bullet,\text{max}} - i_{\bullet,\text{min}}}, \tag{1}$$

were we define $n(i_\bullet)$ as the **saturation map function**. These minimum and maximum intensities are derived experimentally by exposing the cell to the maximum possible irradiance intensity until no further export was observed. This step corresponds to the *calibration* step mentioned on the user guide in Sec. 1.7.

It was found that cytosolic intensity measurements were sensitive to experimental conditions such as focal plane, segmentation accuracy and cell shape. Consequently, controller design is done using the nucleic intensity, under the assumption that in the saturation reference frame the controlled response should behave identically (in reverse sensitivity).

3.1 System modelling and identification

The four different states of the LEXY construct led to a four-compartment model, presented in Supplementary Figure 14a. In the figure, n and c are respectively the concentrations in the nucleus and cytosol, with sub-indexes i and a showing (optogenetically) inactive or active proteins, respectively. These four types of concentration represent four states, with specific transition rates k between them. Additionally, the term u represent the irradiance of the optogenetic light, affecting the transfer rate between active and inactive states.

The transition rates k_d, k_{ex} and k_{im} are constant, whilst we assume a linear relationship of illumination intensity u on the activation rates k'_n and k'_c, $k'_n = k' + k_n u$ and $k'_c = k' + k_c u$. Experimentally, we found that the nucleic and cytosolic intensities increased uniformly over time, indicating that diffusion dynamics are of a different time-scale compared than the transition rates between states, allowing us to model the dynamics as reaction rate equations.

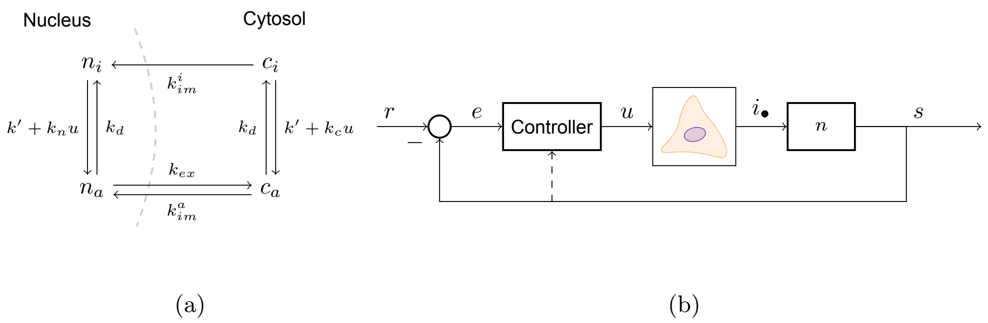

Supplementary Figure 14: **The four-compartment model of LEXY cells.** **a)** The four-compartment model of light-induced nucleocytoplasmic transport with transition rates. **b)** Block diagram of signal propagation, showing how a given target saturation setpoint r is controlled using measured intensities $i_\bullet$ using a PID controller, with the saturation map n.

Combining the rate equations with a linear measurement model for the intensity for the nucleocytoplasmic concentrations, leads to

$$\begin{bmatrix}\dot{n}_a\\ \dot{n}_i\\ \dot{c}_a\\ \dot{c}_i\end{bmatrix} = \underbrace{\begin{bmatrix}-k_d-k_{ex} & k' & k^a_{im} & 0\\ k_d & -k' & 0 & k^i_{im}\\ k_{ex} & 0 & -k_d-k^a_{im} & k'\\ 0 & 0 & k_d & -k'-k^i_{im}\end{bmatrix}}_{A}\begin{bmatrix}n_a\\ n_i\\ c_a\\ c_i\end{bmatrix} + u\underbrace{\begin{bmatrix}0 & k_n & 0 & 0\\ 0 & -k_n & 0 & 0\\ 0 & 0 & 0 & k_c\\ 0 & 0 & 0 & -k_c\end{bmatrix}}_{B}\begin{bmatrix}n_a\\ n_i\\ c_a\\ c_i\end{bmatrix},$$
$$\underbrace{i_n}_{y} = \underbrace{\begin{bmatrix}M_n & M_n & 0 & 0\end{bmatrix}}_{C}\begin{bmatrix}n_a\\ n_i\\ c_a\\ c_i\end{bmatrix}, \tag{2}$$

with M_n the proportional relation between nuclear concentration and fluorescent intensity. Because the dynamics can be arbitrarily scaled, we set $M_n = 1$ to have structural identifiability.

Writing y_s for measurements at time step s, and x for the underlying state, we solve

$$\begin{aligned}\min_{\theta, x_0} \quad & \sum_s \|y_s - \hat{y}_s(\theta, x_0)\|_2\\ \text{s.t.} \quad & \hat{y}_s = f(k, u, \theta, x_0)\end{aligned}$$

using Matlab's nonlinear grey-box system identification tools to find the system parameters $\theta = \{k_d, k_{ex}, \dots\}$ and initial condition x_0 [13, 14]. The function f is the solution of Equation 2 at time step s, given the input sequence u, parameters θ and initial condition x_0. For numerical stability of the optimization problem, the

intensity measurements are normalized to the maximum nucleus intensity, making the maximum nucleic intensity during optimization $\bar{i} = 1$ and the background intensity corresponding to $\underline{i} = 0$. Note that this normalization differs from the saturation map in Expression 1. The coordinate transform leaves the matrices A, B and C unaffected, and the initialization for the parameters in the nonlinear optimization were found heuristically.

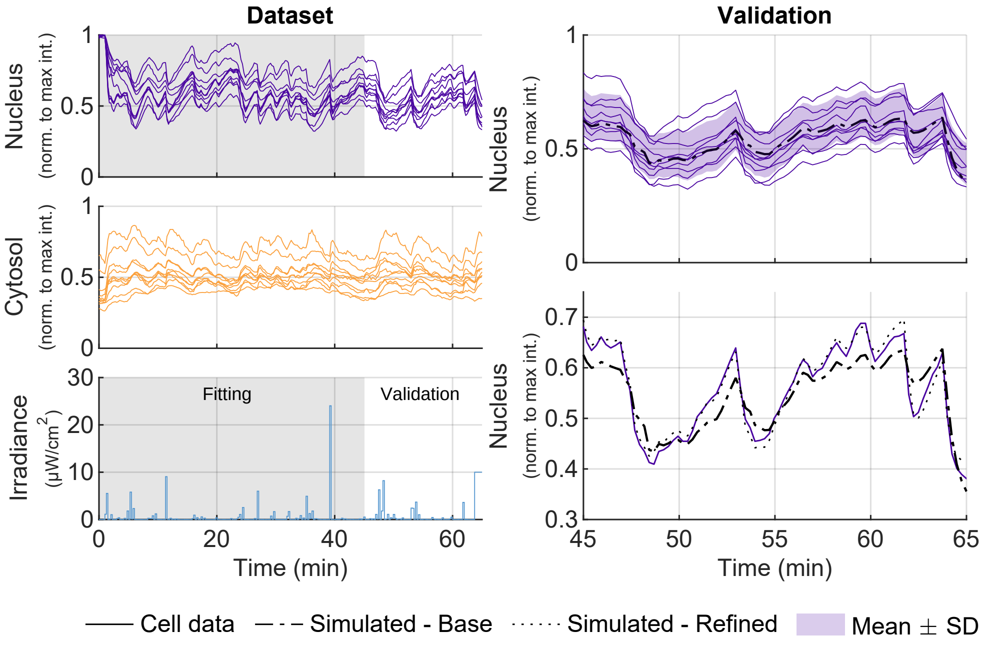

Supplementary Figure 15: **The fitting dataset used for system identification.** Dataset of 10 sequences used for system identification split into fitting (left) and validation (right). For validation, error bands show mean ± standard deviation (SD) of the base model. Bottom right shows a detailed plot of cell data compared to a simulation of its associated refined model. Source data are provided as a Source Data file.

For the fitting data, 10 cells were exposed to a 65-minute sequence of exponentially distributed irradiance intensities ranging from 0 to 25 µW cm^{-2}, taking a measurement every 15 seconds. The measured response is illustrated in Supplementary Figure 15.

Using the validation dataset, the base model resulted in a Variance Accounted For (VAF) of 60 to 80 %, whilst those of the refined models range from 75 to 99 % [15] . A summary of the optimized parameters is presented in Supplementary Table 3.

3.2 Controller design

To design the PID controller, the aim was to achieve minimal overshoot ($< 5\%$) across different cells with a response time in the range of 1-5 minutes. With appropriate

	Fit
k_{ex}	0.2 ± 0.1
k_{im}^{a}	0.03 ± 0.02
k_{im}^{i}	0.05 ± 0.03
k_d	0.004 ± 0.001

	Fit
k'	0.004 ± 0.002
k_n	0.02 ± 0.02
k_c	0.82 ± 0.04

Supplementary Table 3: Mean fitted parameters for the nucleocytoplasmic transport dynamics with their standard deviation.

Nucleic saturation s	K_p	K_i	K_d
0.0	90	0.5	3
0.25	45	0.25	1.5
0.5	9	0.05	0.3
0.75	5	0.05	0.2
1.0	3	0.02	0.2

Supplementary Table 4: Gain-scheduling PID parameters across the operating range for nucleic saturation.

proportional (K_p), integral (K_i) and derivative (K_d) gains, the controller can achieve reference tracking by driving an error e_s to 0 over time. To steer the intensity $i_\bullet$ to a reference r_s, we have $e_s = (i_\bullet)_s - r_s$. In discrete time, the PID controller computes the light intensity u_s,

$$u_s = K_p e_s + K_i \sum_{i \leq s} e_i \ \mathrm{d}t + K_d \frac{e_s - e_{s-1}}{\mathrm{d}t}.$$

The integrator component, $\sum e_i \ \mathrm{d}t$, was bounded from below to -0.1 to mitigate windup due to a bounded input. To find the controller gains satisfying the requirements, the dynamics were linearized around saturation operating points $n(i_\bullet) \in \{0, 0.25, 0.5, 0.75, 1.0\}$. Integral gains were then maximized until the 5% overshoot limit was reached for the standard deviation in the models in frequency-domain. Next, the proportional gains were increased to increase the response time until the same 5% overshoot limit was reached. Stricter overshoot requirements can be obtained using the same method, at the cost of a slower response time.

For the non-gain-scheduled controller, the controller at saturation $n(i_\bullet) = 0.5$ was chosen, whose gains were later adapted slightly to reduce oscillations at low-export conditions. This lead to the parameter set $(K_p, K_i, K_d) = (9.0, 0.005, 0.3)$

Because of the nonlinear dynamics, gain-scheduling was employed to improve the performance at low and high saturation. Different parameters were found at four additional operating points, leading mostly to improved performance at low-saturation conditions. These gains are presented in Supplementary Table 4.

The linearized closed-loop frequency response from reference input to measured output at different operating points is presented in Supplementary Figure 16. Note that the PID does not achieve the 5% overshoot requirement when constraint to the response-time wishes across the saturation range, contrary to gain-scheduling.

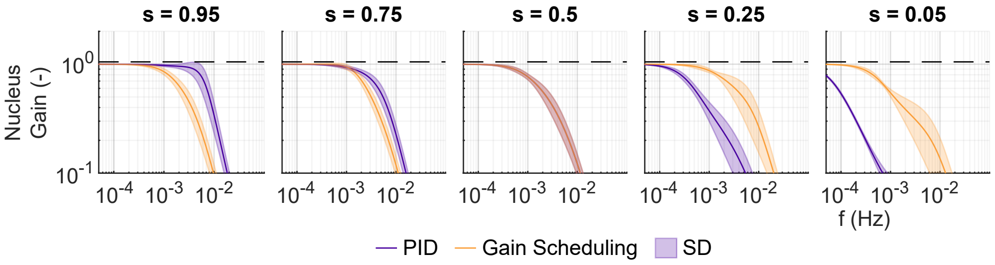

Supplementary Figure 16: **The frequency response from input to output at different operating points.** Mean frequency response from reference setpoint is shown to measured output for linearizations of the identified models. Error bands show mean ± standard deviation (SD). The dashed line indicates the maximum 5 % overshoot limit. Source data are provided as a Source Data file.

Supplementary References

1. Edelstein, A., Amodaj, N., Hoover, K., Vale, R. & Stuurman, N. Computer control of microscopes using µmanager. *Current Protocols in Molecular Biology* **92**, 14.20.1–14.20.17 (2010).

2. Pinkard, H. *et al.* Pycro-manager: open-source software for customized and reproducible microscope control. *Nature Methods* **18**, 226–228 (2021).

3. Lambert, T. Pymmcore-plus: tools for using micro-manager in a pure-python environment (2023). URL https://doi.org/10.5281/zenodo.7703486.

4. Marin, Z. *et al.* Navigate: an open-source platform for smart light-sheet microscopy. *Nature Methods* **21**, 1967–1969 (2024).

5. Casas Moreno, X., Al-Kadhimi, S., Alvelid, J., Bodén, A. & Testa, I. Imswitch: generalizing microscope control in python. *Journal of Open Source Software* **6**, 3394 (2021).

6. Documentation. Tkinter — python interface to tcl/tk (2025). URL https://docs.python.org/3/library/tkinter.html.

7. Sofroniew, N. *et al.* Napari: a multi-dimensional image viewer for python (2023). URL https://doi.org/10.5281/zenodo.3555620.

8. Documentation. Docker: software for container-based application deployment (2025). URL https://docs.docker.com/.

9. He, K. *et al.* *Masked autoencoders are scalable vision learners*, 15979–15988 (IEEE, New Orleans, LA, USA, 2022). URL https://doi.org/10.1109/CVPR52688.2022.01553.

10. Kirillov, A. *et al. Segment anything*, 3992–4003 (IEEE, Paris, France, 2023). URL https://doi.org/10.1109/ICCV51070.2023.00371.

11. Radford, A. *et al. Learning transferable visual models from natural language supervision*, Vol. 139 of *Proceedings of Machine Learning Research*, 8748–8763 (PMLR, 2021). URL https://doi.org/10.5555/3495724.3496161.

12. Ogata, K. *Modern control engineering* Instrumentation and controls series (Prentice Hall, 2010).

13. Ljung, L. *System identification: theory for the user* Prentice Hall information and system sciences series (Prentice Hall PTR, 1999).

14. The MathWorks Inc. System Identification Toolbox: 23.2 (r2023b) (2024). URL https://www.mathworks.com/products/sysid.html.

15. Verhaegen, M. & Verdult, V. *Filtering and system identification: a least squares approach* (Cambridge University Press, 2007).
