# Supplementary material for: Closed-loop optogenetic control of cell biology enables outcome-driven microscopy
Source: Nat Commun. 2025 Dec 23;17:1087. doi: 10.1038/s41467-025-67848-5 (PMC12852791; doi:10.1038/s41467-025-67848-5)
Supplement: Supplementary file 3 — Description of Additional Supplementary Files [file 41467_2025_67848_MOESM3_ESM.pdf]

Description of Additional Supplementary Files

Supplementary Movie 1: Outcome-driven control of the directed cell migration of a HT1080-TIAM cell with constant irradiance.

Supplementary Movie 2: Outcome-driven control of the directed cell migration of a HT1080-TIAM cell with changing irradiance.

Supplementary Movie 3: Simultaneous outcome-driven control of the directed cell migration of multiple HT1080-TIAM cells, with an active avoidance system to prevent collisions.

Supplementary Movie 4: Outcome-driven control of the directed cell migration of a HT1080-TIAM cell in a crowd of non-responsive WT HT1080 cells.

Supplementary Movie 5: Outcome-driven control of the directed cell migration of a transfected MCF7 cell.

Supplementary Movie 6: Outcome-driven control of the nuclear intensity of a U2OS-LEXY cell at full operating range, repeated three times in the same cell.

Supplementary Movie 7: Long-term outcome-driven control of the nuclear intensity of a U2OS-LEXY cell to multiple repeated setpoints.

Supplementary Movie 8: Outcome-driven control of the nuclear intensity of multiple U2OS-LEXY cells with independent irradiance using pulse-width modulation.
