# Supplementary material for: Closed-loop optogenetic control of cell biology enables outcome-driven microscopy
Source: Nat Commun. 2025 Dec 23;17:1087. doi: 10.1038/s41467-025-67848-5 (PMC12852791; doi:10.1038/s41467-025-67848-5)
Supplement: Supplementary file 12 — Reporting Summary [file 41467_2025_67848_MOESM12_ESM.pdf]

nature portfolio

Corresponding author(s): Lukas Kapitein

Last updated by author(s): Nov 26, 2025

Reporting Summary

Nature Portfolio wishes to improve the reproducibility of the work that we publish. This form provides structure for consistency and transparency in reporting. For further information on Nature Portfolio policies, see our Editorial Policies and the Editorial Policy Checklist.

Statistics

For all statistical analyses, confirm that the following items are present in the figure legend, table legend, main text, or Methods section.

n/a	Confirmed	
☐	☒	The exact sample size (*n*) for each experimental group/condition, given as a discrete number and unit of measurement
☐	☒	A statement on whether measurements were taken from distinct samples or whether the same sample was measured repeatedly
☐	☒	The statistical test(s) used AND whether they are one- or two-sided<br>*Only common tests should be described solely by name; describe more complex techniques in the Methods section.*
☒	☐	A description of all covariates tested
☒	☐	A description of any assumptions or corrections, such as tests of normality and adjustment for multiple comparisons
☐	☒	A full description of the statistical parameters including central tendency (e.g. means) or other basic estimates (e.g. regression coefficient) AND variation (e.g. standard deviation) or associated estimates of uncertainty (e.g. confidence intervals)
☐	☒	For null hypothesis testing, the test statistic (e.g. *F*, *t*, *r*) with confidence intervals, effect sizes, degrees of freedom and *P* value noted<br>*Give P values as exact values whenever suitable.*
☒	☐	For Bayesian analysis, information on the choice of priors and Markov chain Monte Carlo settings
☒	☐	For hierarchical and complex designs, identification of the appropriate level for tests and full reporting of outcomes
☒	☐	Estimates of effect sizes (e.g. Cohen's *d*, Pearson's *r*), indicating how they were calculated

Our web collection on statistics for biologists contains articles on many of the points above.

Software and code

Policy information about availability of computer code

Data collection	In this study, our microscope bridge module uses µ-Manager version 2.0 Nightly Builds 20220930 (Nikon Ti) & 20250310 (Nikon Ti-2E) to control the microscopes and peripheral components, and the Pycro-Manager version 0.19.2 package as a translation layer. Custom code for our smart microscopy platform is available on GitHub: https://github.com/UU-cellbiology/UU_SmartMicroscopy/releases/tag/v1.0.0.
Data analysis	All post-experiment imaging processing was performed using FIJI 1.54f. Data processing was performed using Python, GraphPad Prism 9.5.0, or Microsoft Excel. GraphPad Prism 9.5.0 and Adobe Illustrator 28.7 were used for data visualisation. Matlab v.2023b was used for controller design.

For manuscripts utilizing custom algorithms or software that are central to the research but not yet described in published literature, software must be made available to editors and reviewers. We strongly encourage code deposition in a community repository (e.g. GitHub). See the Nature Portfolio guidelines for submitting code & software for further information.

Data

Policy information about availability of data

All manuscripts must include a data availability statement. This statement should provide the following information, where applicable:

- Accession codes, unique identifiers, or web links for publicly available datasets
- A description of any restrictions on data availability
- For clinical datasets or third party data, please ensure that the statement adheres to our policy

All datasets generated and analysed during this study, including raw and processed image files (TIFF), quantitative data (CSV, XLSX), and custom analysis code (Python scripts), are available in the Yoda database: https://doi.org/10.24416/UU01-M0U1OT. Source data are provided with this paper.

Research involving human participants, their data, or biological material

Policy information about studies with human participants or human data. See also policy information about sex, gender (identity/presentation), and sexual orientation and race, ethnicity and racism.

Reporting on sex and gender	N/A
Reporting on race, ethnicity, or other socially relevant groupings	N/A
Population characteristics	N/A
Recruitment	N/A
Ethics oversight	N/A

Note that full information on the approval of the study protocol must also be provided in the manuscript.

Field-specific reporting

Please select the one below that is the best fit for your research. If you are not sure, read the appropriate sections before making your selection.

☒ Life sciences ☐ Behavioural & social sciences ☐ Ecological, evolutionary & environmental sciences

For a reference copy of the document with all sections, see nature.com/documents/nr-reporting-summary-flat.pdf

Life sciences study design

All studies must disclose on these points even when the disclosure is negative.

Sample size	For cell migration studies, n = 58 cells were controlled with various irradiance levels and analysed. For control of nucleocytoplasmic transport, control was demonstrated using n = 4 cells for the standard controller and n = 10 cells for the gain-scheduled controller. Repetitions within the same cell were repeated three times for each controller. For all analyses, no more than two cells per coverslip were used to ensure robustness between sample preparations.
Data exclusions	No data points were excluded from analysis.
Replication	For both cell migration and nucleocytoplasmic transport studies, replication was demonstrated by (i) controlling multiple cells to the same migration path (e.g. Fig.1e) or cytosol value (Fig. 3c), and (ii) repeating control in the same cell (Fig. 1c and Fig. 3d).
Randomization	Randomisation was not necessary in this study due to the lack of distinct experimental groups.
Blinding	Blinding was not necessary in this study due to the lack of distinct experimental groups.

Reporting for specific materials, systems and methods

We require information from authors about some types of materials, experimental systems and methods used in many studies. Here, indicate whether each material, system or method listed is relevant to your study. If you are not sure if a list item applies to your research, read the appropriate section before selecting a response.

Materials & experimental systems

n/a	Involved in the study	
☐	☒	Antibodies
☐	☒	Eukaryotic cell lines
☒	☐	Palaeontology and archaeology
☒	☐	Animals and other organisms
☒	☐	Clinical data
☒	☐	Dual use research of concern
☒	☐	Plants

Methods

n/a	Involved in the study	
☒	☐	ChIP-seq
☐	☒	Flow cytometry
☒	☐	MRI-based neuroimaging

Antibodies

Antibodies used	Cells were incubated with primary antibody (rabbit anti-RFP; 1:500; Rockland, 600-401-379) for 2 hours at room temperature, followed by washing and secondary antibody incubation (goat anti-rabbit Alexa Fluor 488; 1:1000; Thermo Fisher Scientific, A-11034) for 1 hour at room temperature.
Validation	anti-RFP was validated by the manufacturer (Rockland). "Antibody was prepared from monospecific antiserum by immunoaffinity chromatography using Red Fluorescent Protein (Discosoma) coupled to agarose beads followed by solid phase adsorption(s) to remove any unwanted reactivities. Expect reactivity against RFP and its variants: mCherry, tdTomato, mBanana, mOrange, mPlum, mOrange and mStrawberry. Assay by immunoelectrophoresis resulted in a single precipitin arc against anti-Rabbit Serum and purified and partially purified Red Fluorescent Protein (Discosoma). No reaction was observed against Human, Mouse or Rat serum proteins."

Eukaryotic cell lines

Policy information about cell lines and Sex and Gender in Research

Cell line source(s)	HT1080 (ATCC, CCL-121), MCF7 (ATCC, HTB-22), U2OS Flp-in T-Rex (a gift from Alessandro Sartori) and COS-7 (ATCC, CRL-1651) cells were used
Authentication	Cells sourced from ATCC were authenticated by ATCC.
Mycoplasma contamination	Cells were routinely checked for mycoplasma and discarded if positive.
Commonly misidentified lines (See ICLAC register)	N/A

Plants

Seed stocks	N/A
Novel plant genotypes	N/A
Authentication	N/A

Flow Cytometry

Plots

Confirm that:

☒ The axis labels state the marker and fluorochrome used (e.g. CD4-FITC).

☒ The axis scales are clearly visible. Include numbers along axes only for bottom left plot of group (a 'group' is an analysis of identical markers).

☒ All plots are contour plots with outliers or pseudocolor plots.

☒ A numerical value for number of cells or percentage (with statistics) is provided.

Methodology

Sample preparation	HT1080-TIAM cells generated by lentiviral transduction and blasticidin selection were harvested, resuspended in DPBS as a single-cell suspension, and FACS sorted.
Instrument	FACSAria™ Fusion Flow Cytometer (BD Biosciences)
Software	Flow cytometry data were collected and analysed using BD FACSDiva 9.4 software (BD Biosciences).
Cell population abundance	Post-sort analysis yielded 48,632 total events, with 1,147 events (2.4%) falling within the double-positive P4 gate. Purity of the sorted fraction was assessed by fluorescence microscopy immediately after sorting.
Gating strategy	Gates were applied to select the primary cell population using Forward Scatter-Area (FSC-A) vs Side Scatter-Area (SSC-A), select singlets using Forward Scatter-Width (FSC-W) vs Forward Scatter-Height (FSC-H) and Side Scatter-Width (SSC-W) vs Side Scatter-Height (SSC-H), and finally select cells positive for both mVenus (530/30, 488 nm laser) and TagRFP-t (582/15, 561 nm laser). Boundaries were manually drawn based on the characteristic density clusters observed in FSC/SSC and fluorescence parameter spaces. Gates were positioned to encompass the dominant cell population while excluding debris, aggregates, and background fluorescence.

☒ Tick this box to confirm that a figure exemplifying the gating strategy is provided in the Supplementary Information.
